# Supplementary material for: Topological states and phase transitions in Sb2Te3-GeTe multilayers
Source: Sci Rep. 2016 Jun 13;6:27716. doi: 10.1038/srep27716 (PMC4904215; doi:10.1038/srep27716)
Supplement: Supplementary Information [file srep27716-s1.pdf]

Supplementary information: Topological states and phase transitions in Sb_2Te_3-GeTe multilayers

Thuy-Anh Nguyen, Dirk Backes, Angadjit Singh, Rhodri Mansell, Crispin Barnes, David Ritchie, Gregor Mussler, Martin Lanius, Detlev Grützmacher and Vijay Narayan*

* vn237@cam.ac.uk

[1] Cavendish Laboratory, Department of Physics, University of Cambridge, J. J. Thomson Avenue, Cambridge CB3 0HE, United Kingdom
[2] Peter Grünberg Institute (PGI-9), Forschungszentrum Jülich, 52425 Jülich, Germany

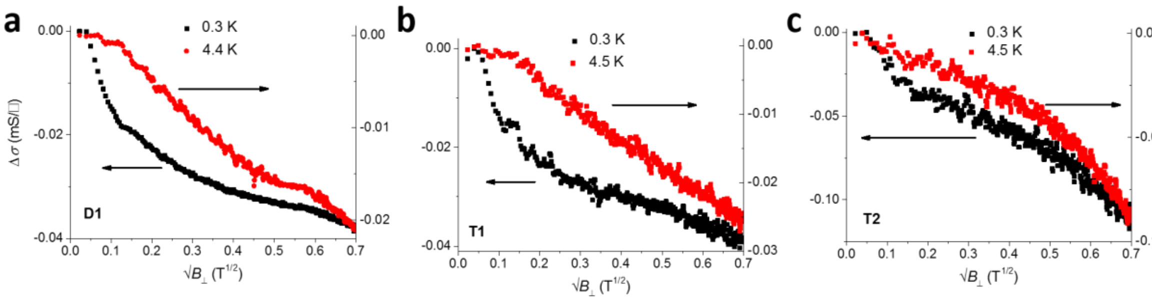

Figure S1. (a) – (c) show the WAL corrections as a function of $\sqrt{B_\perp}$. Clearly, the data are not consistent with bulk WAL where $\Delta\sigma_{xx} \sim B_\perp^{1/2}$. [23] The black and red traces correspond to $\Delta\sigma_{xx}$ measured at 0.3 K and 4.5 K, respectively.

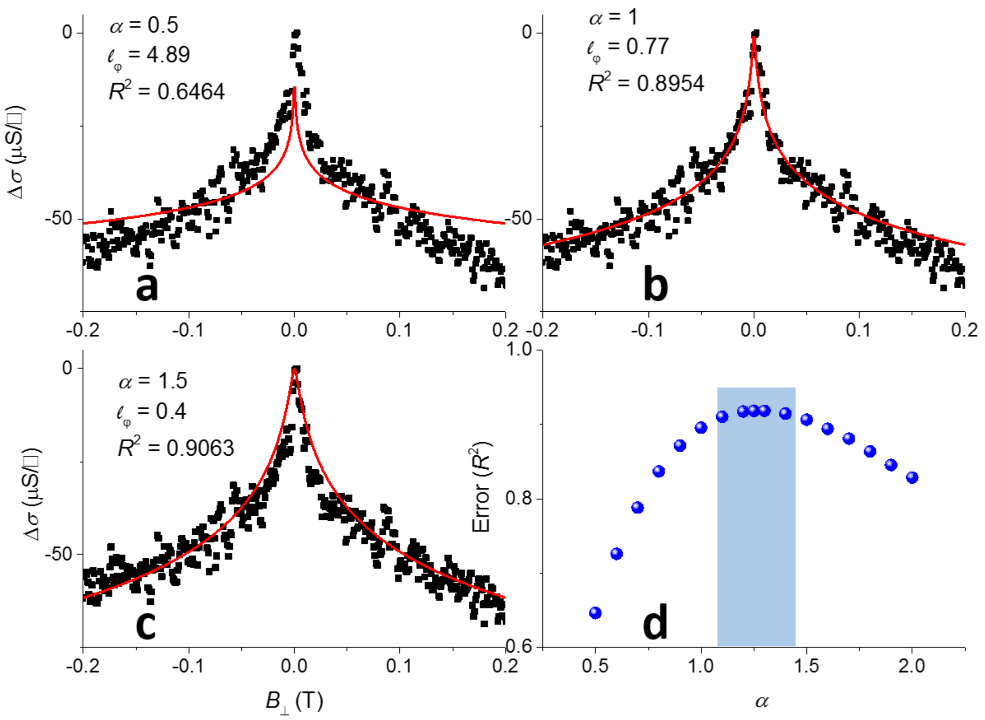

Figure S2. (a) – (c) compare the experimental data (symbols) and HLN fit (solid line) for the fitting parameters listed in each panel. Clearly, $1 < \alpha < 1.5$ yield the best fits, consistent with three TSMs. In order to produce these graphs, α was held constant and ℓ_φ varied to obtain the best fit. The R^2 measure of the fit, i.e., the squared sum of difference between the experimental and theoretical curves was used to gauge the quality of the fit. As is shown in (d), the R^2 measure shows a maximum in the range of $\alpha = 1.25$.
